# The brain’s unique take on algorithms

**DOI:** 10.1038/s41467-023-40535-z

**Published:** 2023-08-16

**Authors:** James B. Aimone, Ojas Parekh

**Affiliations:** Neural Exploration and Research Laboratory, Center for Computing Research, Sandia National Laboratories, Albuquerque, NM USA

**Keywords:** Computer science, Network models

## Abstract

The current gap between computing algorithms and neuromorphic hardware to emulate brains is an outstanding bottleneck in developing neural computing technologies. Aimone and Parekh discuss the possibility of bridging this gap using theoretical computing frameworks from a neuroscience perspective.

## Teaser

Perspectives for understanding the brain vary across disciplines and this has challenged our ability to describe the brain’s functions. In this comment, we discuss how emerging theoretical computing frameworks that bridge top-down algorithm and bottom-up physics approaches may be ideally suited for guiding the development of neural computing technologies such as neuromorphic hardware and artificial intelligence. Furthermore, we discuss how this balanced perspective may be necessary to incorporate the neurobiological details that are critical for describing the neural computational disruptions within mental health and neurological disorders.

Understanding the foundations of the brain’s computation is critically important for both advancing computing and treating neurological conditions. Despite the increasing successes of modern artificial intelligence (AI), biological intelligence remains unmatched and orders of magnitude more energy efficient at many cognitive tasks. Meanwhile, some brain disorders can be viewed as diseases of computation^1^, with our future ability to treat them requiring that we have an ability to manipulate and fix those computations^2^. Understanding the brain’s computations thus addresses two significant needs of modern society – energy efficiency and mental health – but realizing this goal has proven challenging.

Central to this challenge is how neural computing has been approached, whereby different communities have taken either the top-down approach of designing algorithms with modest brain-inspiration, such as artificial neural networks (ANNs), or the bottom-up approach where computing hardware is designed to emulate physical properties of the brain. For instance, in recent years, ANNs and other AI methods, which are largely linear algebra in implementation, have illustrated that many features of the brain can be successfully incorporated into conventional algorithm frameworks. Nonetheless, while ANNs are a fruitful framework to describe the brain’s computation, particularly in sensory systems^3^, there remains an awkwardness of using algorithms optimized for graphics processing units (GPUs)^4^ to efficiently describe the diverse computations of the brain, many of which are not as immediately well-described by current AI^5^. The bottom-up approach to neural computing has made progress in a different direction, with several approaches to neuromorphic hardware prioritizing energy-efficiency by focusing on specific aspects of biological brains, including spiking communication, analog computation, processing-in-memory, and local learning^6^.

Inconveniently, many aspects of neurobiology have yet to be fully realized in either AI algorithms or neuromorphic hardware. Such areas of neuroscience focus include learning across a wide range of spatiotemporal scales, ubiquitous stochasticity, diversity and heterogeneity of neuron types and parameters, neuromodulation, and development^7^. Today’s neuromorphic hardware rarely accounts for this complexity because there are no algorithms that stand ready to take advantage of it, and algorithms similarly do not leverage such complexity because of its inefficiency on today’s hardware. We know from neuroscience that many of these features are critical for understanding human cognition and disease; for instance, neuromodulators such as serotonin and norepinephrine being key to the brain’s flexibility and learning as well as underlying many psychiatric disorders^8^, yet remaining largely unexplored from a computational perspective.

Conveniently, theoretical computer science offers us tools to help resolve some of this uncertainty. Clarifying the distinctions between different approaches to computing is fundamental to computational complexity theory^9^, which broadly seeks to understand the power and limitations of different and often exotic models of computing. Some of the most celebrated results in complexity theory stem from realizing that seemingly disparate models of computing are able to solve precisely the same class of problems. Unexpected characterizations of fundamental models of computing, studied for decades, are still being discovered. For example, problems solvable by Turing machines in polynomial time can be characterized in terms of solutions to certain kinds of ordinary differential equations^10^. Such diversity in perspectives of computation is important, as it critically shapes the way problems are expressed and solved.

**Fig. 1:**
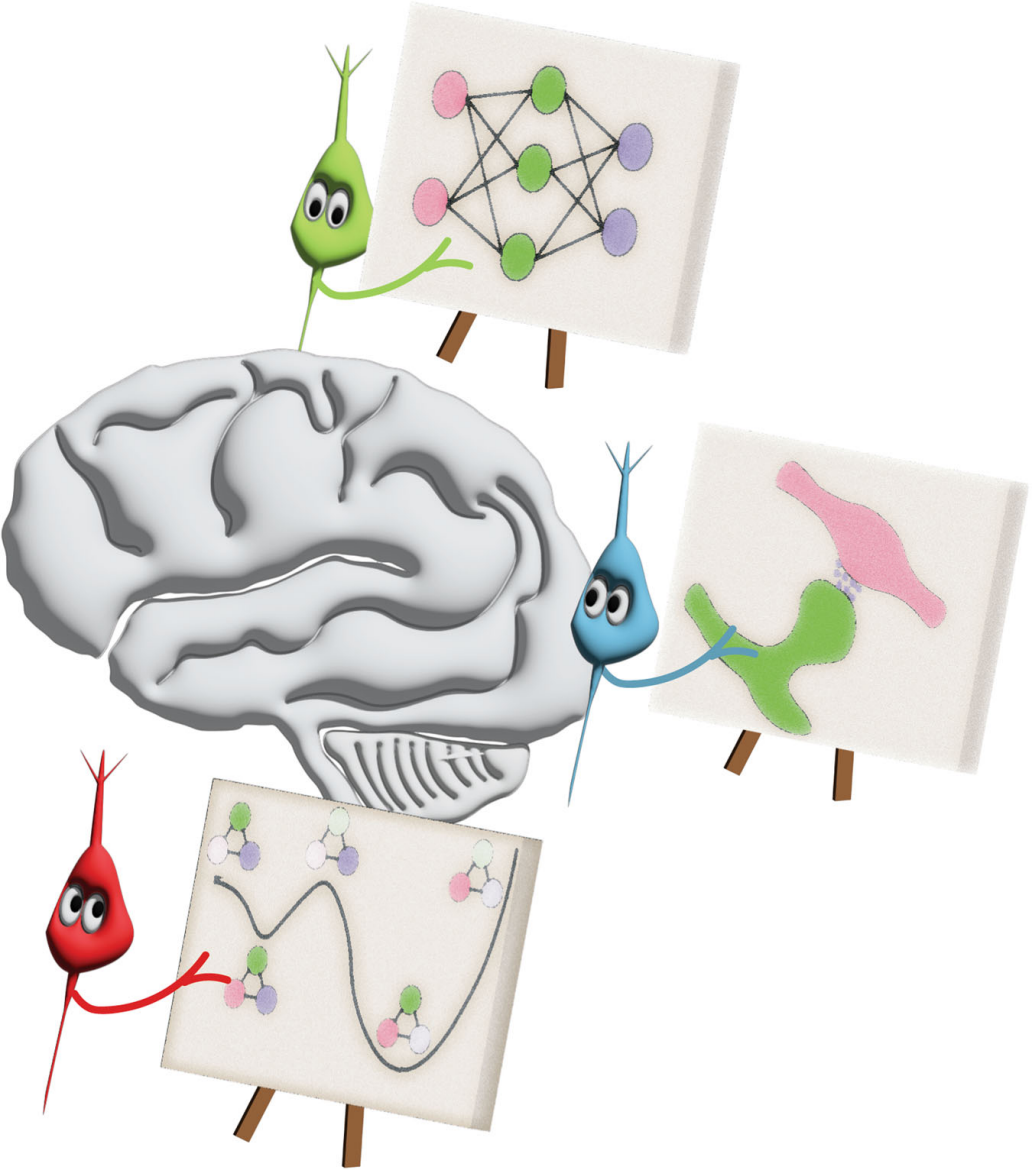
Different disciplines have different perspectives of the brain. Like the parable of the blind men and the elephant, different research communities (illustrated as neurons) often see only what they expect to see in the brain. A computer scientist’s perspective (green neuron) may be biased towards neural networks with established utility, a physicist (red neuron) may seek the energy landscapes that have proven invaluable for other questions, and a neuroscientist (blue neuron) may aim to describe the incredibly complex biology of neural circuits. The pursuit of a common theoretical framework that bridges top-down and bottom-up computational perspectives while allowing the realities of biology to be incorporated will be critical in furthering our understanding of the brain.

Through this view, it is worth stepping back and identifying what the right computational model for neural computing is. While today’s large-scale spiking platforms are Turing complete in a trivial sense, there is a stark difference between the bottom-up features emphasized in neuromorphic hardware and the top-down algorithms used in AI (Fig. 1). This disconnect indicates that perhaps the generic Turing model of computation is not best suited for describing the cognitive functions that emerge from the brain’s physical structure. While there will continue to be debates about whether neuromorphic hardware offers advantages over conventional hardware, we argue that a more productive goal would be to identify an effective abstract model of neural computation. Such an abstract model would allow us to analyze the potential benefits of extending neuromorphic approaches with new features from the biology and a useful model will enable us to design effective programming models that are necessary for its broader use.

Designing computational models for neuromorphic computing presents many unique challenges and pitfalls. For example, a flexible model that highlights the strengths of neuromorphic computing ought to address analog computation; however, realistic continuous models of computing can be challenging to design and may inadvertently introduce abilities to solve otherwise uncomputable problems in finite time^11^. Yet designers of neuromorphic computing models may draw inspiration from successful continuous computational models^10,11^. Neuromorphic computing is also massively parallel and uses asynchronous communication (i.e., there is no global clock), and thus it may be unfair to compare directly to conventional algorithms that do not take communication costs into account^12^. Fair and rigorous comparisons are paramount when considering the potential asymptotic advantages that a neuromorphic computing approach may offer over conventional computing. Consequently, massively parallel or distributed models of conventional computing are likely better candidates for comparison. Framing comparisons using fair metrics is also critical, and measures such as computation per unit of energy or space may be insightful.

In^13^, Jaeger and colleagues present an alternative framework through which to view neural computation. By starting with the fact that the brain is computing in the physics, which are inherently dynamical and spatiotemporal, they recognize that an appropriate model of computation should similarly be based on the processes that can be physically realized and observed. These ideas are not necessarily new: cybernetics as a field has existed since the early days of computing; von Neumann himself questioned whether the serial programming model was well suited to describe brain-like computations^14^. The fluent computing model proposed by Jaeger and colleagues builds on these efforts by combining the practical advantages in composability (i.e., the ability to construct more complex applications out of simpler algorithms) and associations inherent in conventional computing with constraints taken from the to the physical description of a neural system.

Researchers have long debated whether brain-inspired computing should be approached with a computing with algorithms or a computing with physics emphasis. Like Jaeger and colleagues, we choose to interpret this slightly differently. To allow others to program and use a computing framework, it must be interpretable from a top-down perspective, but in order to be implemented, all frameworks need a bottom-up component. What makes the fluent computing approach unique is that its authors have focused on connecting the bottom-up and top-down approaches. By relating dynamics across scales, the proposed fluent computing can ideally achieve the composability that is expected in conventional algorithm design. Perhaps more exciting, this approach should be extendable to the broad range of spatial and temporal dynamics in the brain that are not typically captured in neural computing today.

It will be important to examine how this formal bottom-up perspective on neuromorphic computing can be connected to other more algorithmic perspectives on neuromorphic computing, such as ANNs, characterizing assemblies of neurons^15^ or vector symbolic architectures^16^. These top-down models offer clear connections to algorithm design and comparisons to conventional computing, but in doing so they potentially subtract away much of the physics that make brains computationally powerful. Arguably, the universality of these top-down models also makes it too easy to dismiss the potential contributions of deeper neural inspiration. By construction, these models can solve almost anything, so why consider the complexities introduced by neurobiological concepts such as modulation or learning? Brains must exist with space and energy constraints, much like the computers and AI systems that we seek to develop. Connecting these frameworks to physical models of computing may provide a mechanism for fairly evaluating what this complexity offers, as well as further providing a path to understanding what it means when these mechanisms are disrupted in injury and disease.

Stepping back, challenging the Turing model of computation as the most effective model to describe neural computation is more than just a philosophical question for theoretical computer science. It brings up a more fundamental question for the neuroscience field in general. After our decades-long pursuit to describe the brain explicitly or implicitly through the lens of Turing computation and von Neumann architectures, it may be worth asking whether we have lost sight of what makes the brain special. Perhaps we have unknowingly abstracted away the very things that we need for understanding cognition and intelligence, and in the process unintentionally handcuffed ourselves in our pursuit of the brain for the purposes of efficient computing and improved health. “Does the brain use algorithms?” is not the right question. The right question is, “Are we even positioned to understand what a neural algorithm even is?”
